# Surface Acoustic Wave-Driven Enhancement of Enzyme-Linked Immunosorbent Assays: ELISAW

**DOI:** 10.1021/acs.analchem.4c01615

**Published:** 2024-05-30

**Authors:** Lei Zhang, Shuai Zhang, Cécile Floer, Sreeya Anjana Raj Kantubuktha, María José González Ruiz Velasco, James Friend

**Affiliations:** † Medically Advanced Devices Laboratory, Center for Medical Devices, Department of Mechanical and Aerospace Engineering, Jacobs School of Engineering, and the Department of Medicine, School of Medicine, University of California San Diego, 9500 Gilman Drive MC0411, La Jolla, California 92093, United States; ‡ Université de Lorraine, Centre national de la recherche scientifique (CNRS), Institut Jean Lamour, F-54000 Nancy, France; ¶ Materials Science and Engineering Program, University of California San Diego, 9500 Gilman Drive, La Jolla, California 92093, United States

## Abstract

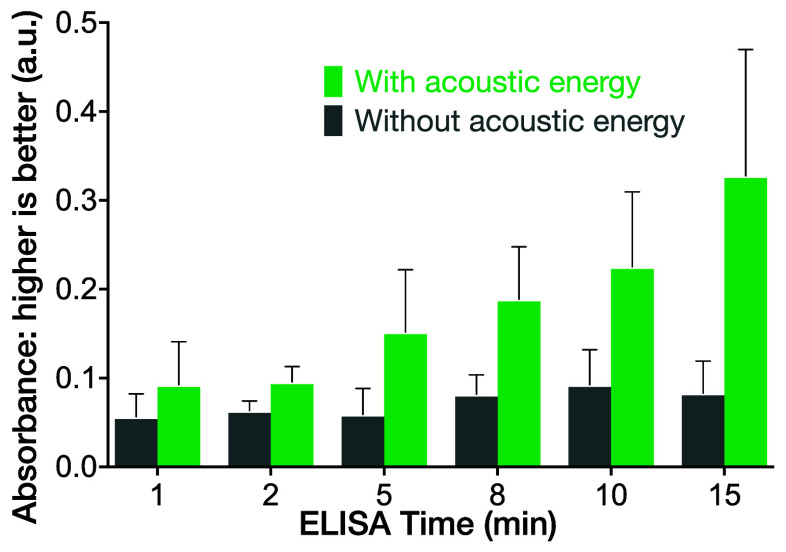

Enzyme-linked immunosorbent assays (ELISAs) are widely used in biology and clinical diagnosis. Relying on antigen–antibody interaction through diffusion, the standard ELISA protocol can be time-consuming, preventing its use in rapid diagnostics. We present a time-saving and more sensitive ELISA without changing the standard setup and protocol, using surface acoustic waves (SAWs) to enhance performance. Each step of the assay, from the initial antibody binding onto the walls of the well plate to the target analyte molecules’ binding for detection—except, notably, for the blocking step—is improved principally via acoustic streaming-driven advection. Using SAWs, the time required for one step of an example ELISA is reduced from 60 to 15 min to achieve the same binding amount. By extending the duration of SAW exposure to 20 min, the sensitivity can be significantly improved over the 60 min, 35 °C ELISA without SAWs. It is also possible to confer beneficial improvements to bead-based ELISA by combining it with SAWs to further reduce the time required for binding to 2 min. By significantly increasing the speed of ELISA, its utility may be improved for a wide range of point-of-care diagnostics applications.

## Introduction

Assays are common across many disciplines, including biology,^1,2^ chemistry,^3,4^ clinical diagnostics,^5^ environmental monitoring,^6,7^ and pharmaceutical research.^8^ Most are designed to detect and quantify proteins, nucleic acids, hormones, enzymes, and similar biomolecules in addition to nonbiological entities like toxins and environmental contaminants. The detection principles vary according to the target. Polymerase chain reaction (PCR) assays^9,10^ are the best known among them. Though very popular, PCR assays solely serve to detect deoxyribonucleic acid (DNA). Surface plasmon resonance (SPR)^11^ and isothermal titration calorimetry (ITC)^12^ assays identify the interaction between two or more molecules, such as receptor–ligand binding or protein–protein binding. There are also assays that involve the separation of analytes based on size, charge, or other properties, such as the Western blot (WB).^13^ Among all these assays, the immunoassay stands out as one of the most frequently used methods because of its versatility, sensitivity, scalability, cost efficiency, and ease of use.^14^ Immunoassays take advantage of the specific interaction between biological reagents, typically antigens and antibodies, to selectively detect the presence and concentration of target analytes in complex biological mixtures.

### Immunoassays

The detection and quantification of target molecules at low concentrations have historically been a significant challenge in immunoassay development. Indeed, Sheehan and Whitman show from calculations that without directed transport of biomolecules, individual nanoscale sensors will be limited to picomolar-order sensitivity for practical time scales.^15^ Sensing low concentrations is crucial, as many biological and clinical applications require the measurement of analytes present at trace levels, ideally in a simple and fast protocol. Building upon competitive binding principles from the early 1950s, Berson and Yalow^16^ developed the radioimmunoassay (RIA) by introducing radioactively labeled insulin into a plasma sample, enabling indirect determination of the unlabeled insulin concentration through competition for antibody binding and subsequent radioactivity measurement. The development of RIA revolutionized clinical diagnostics and research by providing a highly sensitive, specific, and reproducible method for measuring low concentrations of analytes in complex biological samples.^17^ However, the use of radioactive isotopes raised concerns about safety,^18^ prompting a search for alternative labeling methods and detection techniques. The enzyme-based immunoassay (ELISA) was developed in response;^19^ besides offering a nonionizing radiation-based assay, it also provided a longer shelf life than RIA. The ELISA employs enzyme-linked reactions to amplify the detection signal, making it both highly sensitive and specific. Fluorescent immunoassays (FIA)^20^ and chemiluminescent immunoassays (CLIA)^21^ are also popular assays today, detecting analytes through fluorescent labeling and light emission from the chemical reactions, respectively. However, ELISA offers a simple and easy-to-read colorimetric output for quantified detection of the target analyte.

Enzyme-based immunoassays can be used to detect a wide range of analytes, including proteins, antibodies, and hormones.^22^ Compared to lateral flow immunoassays (LFIA) that have been commonplace since the 1970s in their utilization for pregnancy tests^23^ and most recently the near-ubiquitous use in home diagnosis of COVID,^24^ ELISA offers far greater sensitivity and reproducibility with the same general assay conditions and reagents. In good work by some research groups,^25,26^ the sensitivity of LFIA has been improved to nearly compete with ELISA—under certain conditions and for specific assay targets. In the past few years, CRISPR-based (CAS-12/CAS-13a) assays have become enormously popular,^27^ though there remain important questions as to their actual sensitivity.^28^ The wide range of concentration that may be repeatably quantified using ELISA is another reason it is a popular assay, as is the fact it can be performed using parallel, high throughput methods using microplate readers and automated processing equipment.^29,30^ While PCR is another popular method for DNA detection that is likewise automated in many applications, the cost of ELISA is significantly lower: PCR analysis requires specialized reagents, such as Taq polymerase and dNTPs, and specialized equipment, such as thermal cyclers. Though researchers have made progress in lowering the cost and effort of PCR^31−33^ through microfluidics, ELISA remains less expensive.

### Microfluidics Has Improved Assay Cost and Quality

More generally, microfluidics has had a beneficial impact in assay quality and cost reduction, reducing sample sizes and reagent consumption, and reducing the time and effort required in completing assays.^34^ The adoption of soft, easily sealed, and rapidly reproducible materials (polydimethylsiloxane, PDMS)^35^ and paper-based methods^36^ have continued this trend toward better assays for lower costs. For the same reasons, miniaturization techniques have also been improved, thus allowing the integration of micropumps and micromixers into devices.^37−39^ More recently, acoustofluidics has helped to overcome one of the key remaining problems in microfluidics used for lab-on-a-chip applications: the propulsion^40^ and manipulation of fluids and suspended objects within.^41^ There have been demonstrations of its use with PDMS^42^ and paper,^43^ and a number of groups have spent the past decade expanding their understanding^44−46^ and creative use^41,47−49^ of the phenomena.

However, one problem that microfluidics and acoustofluidics have not helped with—at least in ELISA—is its *slowness*. It takes from 3 to 12 h to complete an ELISA: every binding step from the initial antibody binding to the target analyte binding is time-consuming. For this reason, LFIA is also far more common: there are less sensitive versions of LFIA that only require 15 min.^24^ If ELISA was faster, it might provide a much better test for the detection of disease without the drawbacks of having to wait so long^50^ and reducing the user’s worry about potential false positive or negative results.^51^

### Acoustofluidics

One of the attractive aspects of acoustofluidics, in particular, is its ability to significantly speed up phenomena that rely on diffusion. For example, the charging of batteries relies on the diffusion of lithium ions within the electrolyte to replace those ions deposited on the anode and lost from the electrolyte; diffusion is the rate-limiting phenomenon preventing faster charging. Acoustic streaming-driven convection was discovered to convect lithium ions and overcome diffusion limitations, thereby allowing complete and rapid recharging of batteries in only minutes.^52,53^ The analytes in ELISA likewise must diffuse in order to produce the binding necessary for the assay to complete, again representing the rate-limiting phenomenon preventing improvements in the speed of the assay in every binding step. More generally, surface acoustic waves have already been used for mixing purposes in biosensors, in particular to speed up the process.^54,55^

### Surface Acoustic Wave-Enhanced ELISA

Here, we report a SAW-enhanced ELISA, using acoustic streaming to drive advection of the binding and target analyte molecules in each step of the assay. The goal is to accelerate the protein binding to decrease the ELISA time. We also explored how the SAW could greatly increase the sensitivity of the reaction. There is research reporting the use of SAW for detection improvement, however, they are all done with droplets or PDMS channels directly on the transducer which is inconvenient for application.^56,57^ Here we use the 96-well plate which is widely used in ELISA. The experimental setup and protocol are provided in the following sections. Results achieved with the enhanced device are always compared to those obtained with the standard ELISA process.

## Experimental Section: Device Design, Fabrication, Testing, and Assays

### SAW Device Fabrication and Operation

**Figure 1:**
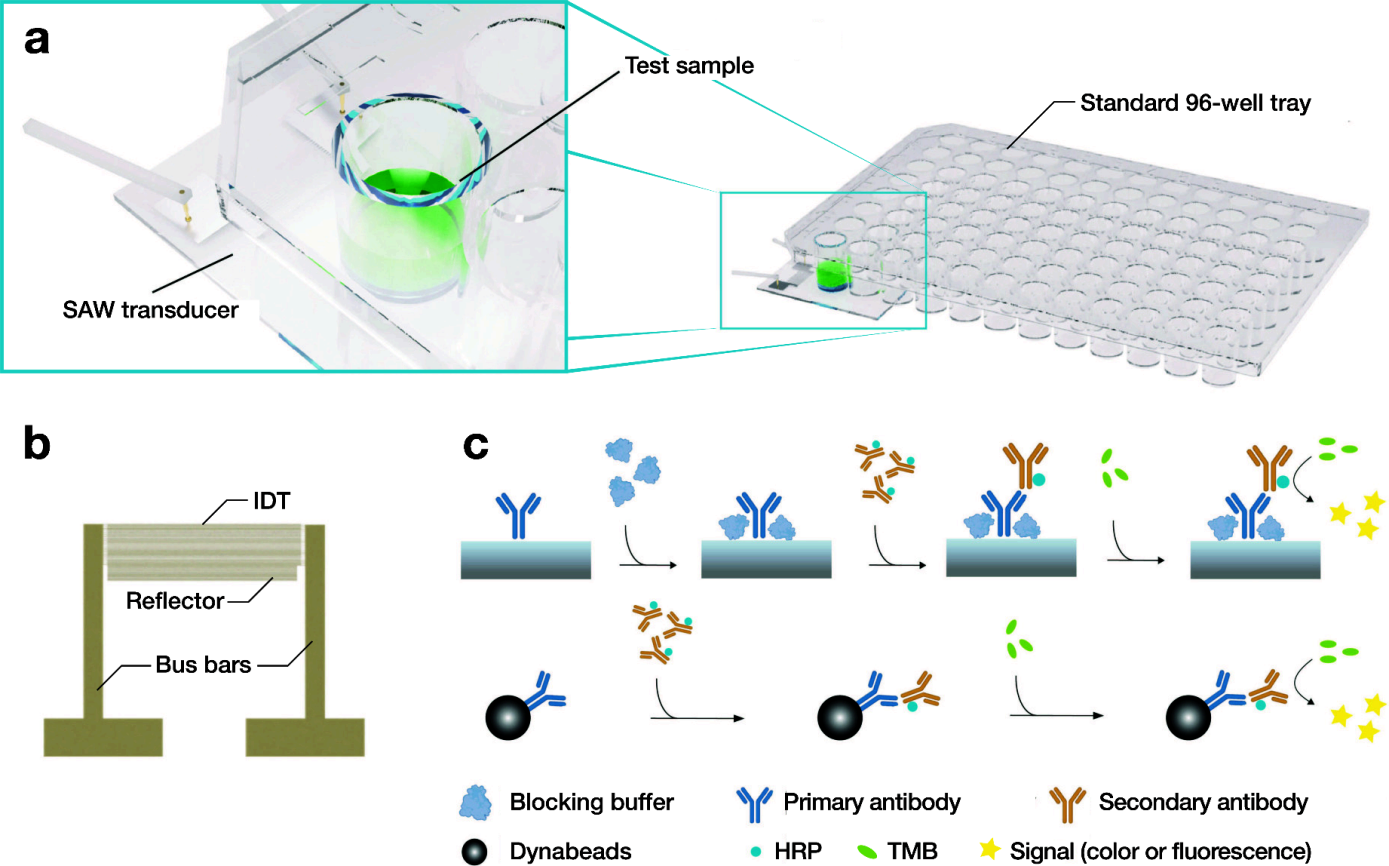
a) Overview of the experimental setup. The test well is 6 mm in diameter containing 200 μL of reagent solution, filling the well to a 6 mm depth. b) Top view of the IDT structure with 24 finger pairs for the IDT and 48 fingers for the reflector distant from the well side one side (here working at 60 MHz). c) The complete ELISA protocol used in this study; the SAW was used in the introduction of the primary antibody, secondary antibody, and analyte. The reason it was not used for the introduction of the blocking buffer is explained in the results.

Double-side polished 127.68° Y-rotated cut lithium niobate (LiNbO_3_) with a thickness of 500 μm was used as a substrate for the SAW device. A single port resonator with a wavelength of λ = 100 μm, 65 μm, or 40 μm (i.e., *f* = 40, 60, or 100 MHz, respectively) was fabricated upon it to generate Rayleigh SAW. Individual 400 nm-thick aluminum interdigital transducers (IDTs) were patterned with 24 finger pairs and 48 reflectors on one side using a standard lithography and lift-off process.^58^ The Rayleigh SAW was generated by applying a sinusoidal electric signal to the IDTs at the resonance frequency using a signal generator (WF1967 200 MHz single channel multifunction generator, NF Corporation, Yokohama, Japan) and an amplifier (403LA, Electronics & Innovation, Ltd., Rochester, NY, USA) at up to 0.8 W of power. The 96-well plate was laid on top of the substrate and the wave was transmitted to the liquid inside the well thanks to a 5 μL acoustic gel droplet placed between the substrate and the plate (*see*Figure 1).

### SAW Device Design

To produce superior mixing from the acoustic streaming, the acoustic wave in the fluid should be attenuated over a length scale comparable to the depth of the fluid sample in order to avoid reflection from the distal boundary, thus forming a traveling wave in support of bulk acoustic streaming from the source. The ideal frequency to use for the SAW may be typically determined by equating the attenuation length of the acoustic wave to the size of the fluid sample to be manipulated. Here, the reagent depth in each of the 96 wells of the tray was found to be 6 mm after introducing the requisite fluids (explained in the following section). Using an expression for the frequency according to this condition from our past work,^52^
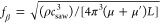
1where ρ is the fluid density, *c*_saw_ is the speed of the surface acoustic wave in the lithium niobate substrate as 3900 m/s, *L* = 6 mm is the length scale over which the acoustic wave needs to be attenuated, and μ and μ′ are the bulk and shear viscosities for the fluid, respectively. Using this equation, the approximate frequency was identified to be 100 MHz.

However, a key limitation of the simplistic eq 1 is the fact it presumes there is no other media along the acoustic wave’s propagation path. Here, there is also 1 mm of polystyrene plastic increasing the attenuation, thus likely reducing the suitable frequency from the estimate produced from eq 1. The length scale should be longer to compensate for the presence of the attenuating plastic. With this in mind, we examined two additional frequencies, 40 and 60 MHz, in addition to the 100 MHz predicted by the equation, and measured the fluid velocity in the well induced by SAW at the same amplitude in the substrate at each of these three frequencies. The particle velocity of the lithium niobate substrate was measured via scanning laser Doppler vibrometry (UHF–120, Polytec, Waldbrönn, Germany) and set to 96 mm/s for all three frequencies by adjusting the input signal amplitude.

**Figure 2:**
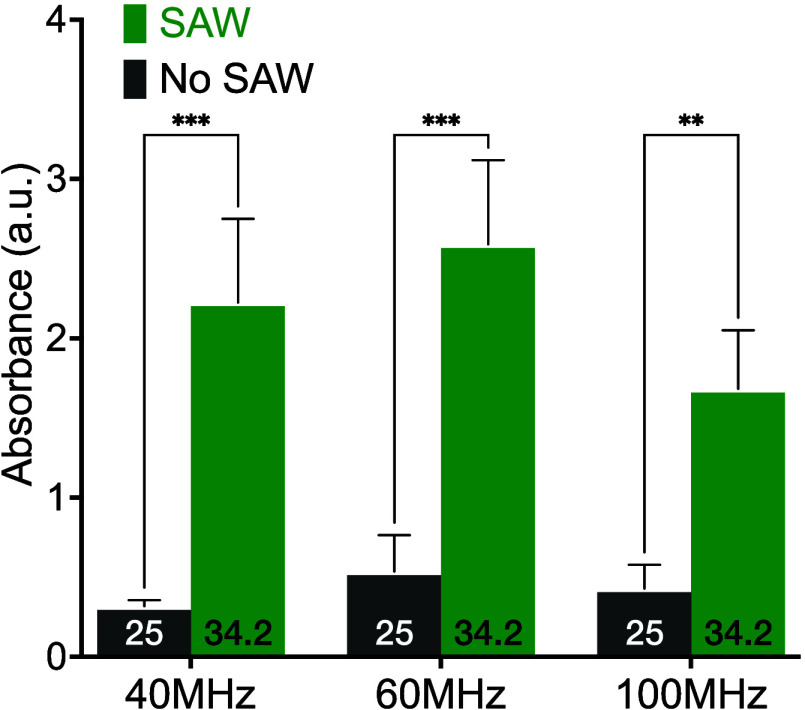
Binding efficiency in terms of light absorbance plotted with respect to the application of 40, 60, and 100 MHz SAW at a particle velocity of 96 mm/s (see Experimental Section: Device Design, Fabrication, Testing, and Assays). The 96-well plates were coated with 200 μL of the goat antichicken IgY-HRP (250 ng/mL) in PBS with SAW for 15 min. Subsequently, TMB was incubated in the well for 5 min without SAW. The numbers at the bottom of each bar are the maximum temperature in the sample, in Celsius, during each run; with SAW, the temperature increased from 25 to 34.2 °C in 15 min. For the no-SAW condition, the ELISA was run at 25 °C from the start. Error bars represent the standard deviation of 6 independent trials. All data was confirmed to be normally distributed via the Shapiro-Wilk test; **p < 0.01, ***p < 0.001.

We then used 0.2 μm fluorescent microspheres (Fluoresbrite© YG Carboxylate Microspheres 16592–1, Polysciences, Warrington, PA, USA) to track the flow while operating the SAW device at 96 mm/s at 40, 60, or 100 MHz using a high-speed camera (Fastcam Mini UX100, Photron, San Diego, CA, USA). These particles are sufficiently small to avoid direct acoustic forces upon them.^59^ According to the average fluid flow velocity given by PIVlab, the flow velocity in the well provided by the 40, 60, and 100 MHz devices were 0.009, 0.004, and 0.0002 m/s, respectively. This illustrates the effect of the attenuating plastic along the acoustic wave propagation path: 40 and 60 MHz acoustic waves produced streaming flows an order of magnitude larger than the 100 MHz choice. Most important, however, is the effect of frequency choice on the binding of an analyte on the wall of the well. To test this, we added 200 μL goat antichicken IgY-HRP solution (immunoglobulin Y with horseradish peroxidase diluted in PBS, ab7118, Abcam, Waltham, MA, USA) to the well, then applied the SAW at 40, 60, or 100 MHz for 15 min, before finally adding TMB (3,3′,5,5′ tetramethylbenzidine, ab171522, Abcam) to incubate for 5 min (with or without SAW). Plotted in Figure 2, the absorbance intensity with and without SAW indicates the effectiveness of the binding at the three frequencies. It appears that 60 MHz is superior to the other two frequency choices, though a check of the data using two-way ANOVA (Analysis of Variance, *p* = 0.141) shows no significant difference between 40 and 60 MHz. In the work that follows, we selected 60 MHz since it shows slightly superior performance overall.

### SAW-Based ELISA

The binding assay followed a general ELISA protocol, with each well in a 96-well plate coated with 200 μL of antiprotein A antibody^60^ (5 μg/mL; ab19483, Abcam, Waltham, MA, USA) in phosphate-buffered saline (PBS, ThermoFisher Scientific, Waltham, MA, USA) for 1 h at 35 °C, washed three times with PBS, then blocked with 200 μL of 1X blocking buffer (ab126587, Abcam) in PBS for 1 h at 35 °C and again washed three times with PBS. Then, a 200 μL goat antichicken IgY-HRP solution was added to each well. Some of the wells were mixed using SAW over a range of times from 1 to 20 min. The no-SAW control wells were left at room temperature (25 °C) in the same well tray for the same period of time. The goat antichicken IgY-HRP solution was then removed after the mixing and the wells were washed three times with PBS. To achieve a quantitative characterization of the binding through a color change, 100 μL of TMB solution as a suitable reactant with HRP was added into the well. After 10 min incubation, 100 μL stop solution (ab171529, Abcam) was added in the well to stop the reaction. The optical density results were measured at a wavelength of 450 nm with a microplate reader (BioTek Synergy H1, Agilent, Santa Clara, CA, USA). The background absorbance was subtracted from all reported values.

For bead-based ELISA, we introduced uniform ϕ2.8 μm superparamagnetic Dynabeads with recombinant Protein A (∼45 kDa) covalently coupled to their surface (ThermoFisher Scientific). The well was first pretreated with 200 μL 1X blocking buffer diluted in PBS for 1 h at 35 °C and then washed three times with PBS. This step was not explicitly required by the protocol but was used to avoid background noise. Then 2.5 μL of Dynabeads (30 mg/mL) was then added into the well and washed with 50 μL of PBS. The PBS was removed by using a magnet to attract the Dynabeads onto a wall of the well. Then 200 μL antiprotein A antibody (5 μg/mL) was added to incubate with the Dynabeads while being rotary mixed at 300 rpm and 26 °C for 10 min (ThermoMixer C, Eppendorf, Enfield, CT, USA). Placing the magnet near the well once again to trap the magnetic microparticles, the supernatant with unbonded antibodies was poured out and the beads and well were washed thrice with PBS. Subsequently, 200 μL goat antichicken IgY-HRP (10 ng/mL) in PBS was then added to each well. One half of the wells were exposed to SAW from 2 to 10 min; the remaining wells were left unexposed to SAW as a control. All the wells were then washed three times in PBS, followed by the addition of 100 μL TMB solution to incubate the system for 10 min without SAW and to create the color change. Finally, 100 μL of stop solution was used to stop the reaction.

## Results

### Effect of SAW on Antibody–Antigen Binding in Traditional ELISA

To test the effect of the Rayleigh SAW on the antigen–antibody binding, we immobilized the antiprotein A antibody in the wells of the 96-well plate and added 200 μL of goat antichicken IgY-HRP at 10 ng/mL. The 60 MHz, 0.8 W, 192 mm/s SAW was applied at this step to mix the solution for a duration of 1 to 15 min, producing both mixing and heating. While the heating does affect the antigen–antibody binding, the temperature increase generated by SAW is less than 35 °C after being applied for 20 min. Notably, the traditional protocol heats the wells to 35 °C for 1 h, and so heating is an inherent part of the protocol.

**Figure 3:**
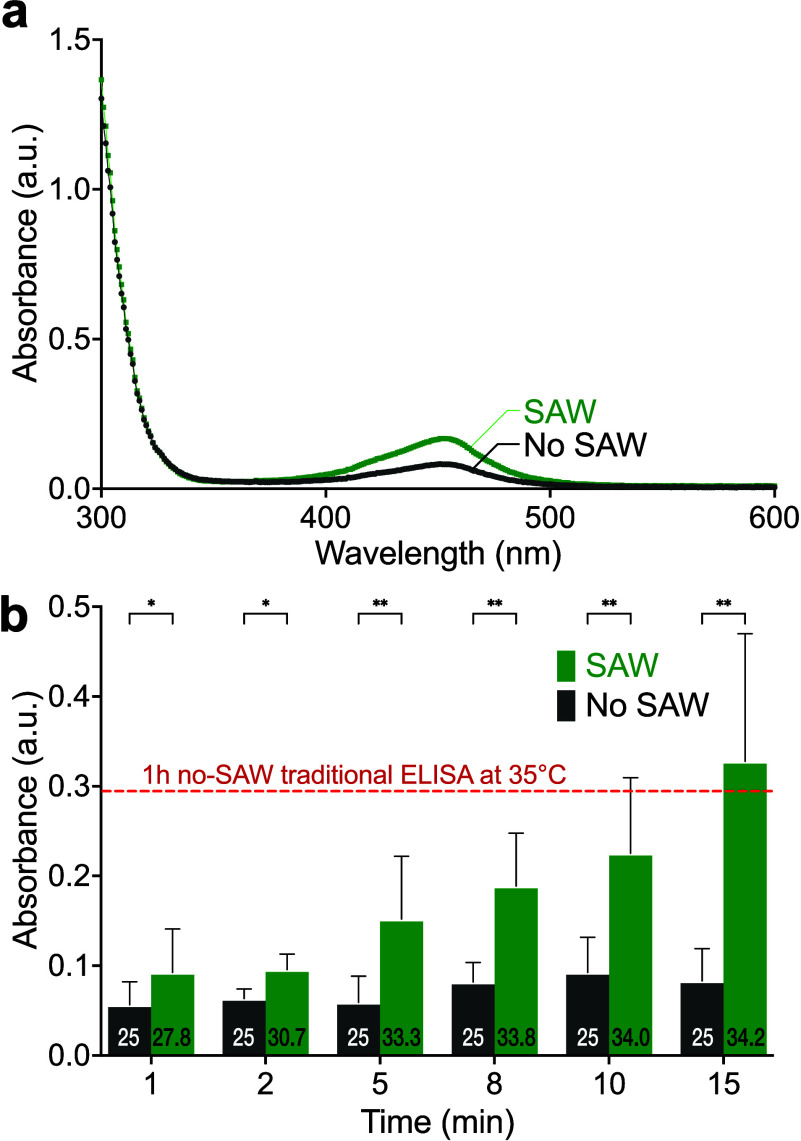
a) Absorbance plotted with respect to wavelength after 1 min with and without SAW already indicates a difference at about 450 nm, with the SAW producing a greater absorbance value. Repeating the (60 MHz, 0.8 W, 192 mm/s) SAW and no-SAW (control) protocols six times for 1 to 15 min describes b) the effect of time on the ELISA protocol. For these cases, there was no externally applied heating, and the temperature of the samples was at the lab temperature of 25 °C, initially for the SAW-driven samples, and throughout for the no-SAW (control) samples. The application of SAW also heats the samples, as indicated by the temperature values provided at the bottom of each bar. However, the heating is generally less than the standard protocol: heating of the sample to 35 °C. As a basis for comparison, the dotted red line represents the absorbance via the standard ELISA protocol of 1 h at 35 °C without SAW. Error bars represent the standard deviation of 6 independent trials. All data was confirmed to be normally distributed via the Shapiro-Wilk test; *p < 0.05, **p < 0.01.

The solution develops a blue color in response to the binding of the secondary antibody to the first, precipitating a reaction of HRP with the added TMB. By adding the stop solution, the enzyme reaction was stopped and turned color from blue to yellow. The strength of the coloration, quantified by the absorbance of light passing through the sample in the wavelength of 450 nm, defines the extent of binding in the sample. Figure 3(a) shows an example of the absorbance curves obtained after 1 min of goat antichicken IgY-HRP incubation in the well. The maximum intensity was 0.083 and 0.168 for the control (no-SAW) and SAW-driven samples, respectively. Figure 3(b) plots the absorbance of the samples after an elapsed time of 1 to 15 min with and without SAW. The horizontal dashed line indicates the absorbance from ELISA after 1 h without SAW at 35 °C in this step. After 1 or 2 min, the binding efficiency indicated by the absorbance value is poor with or without SAW, but nonetheless using SAW significantly (*p* < 0.05) increases the absorbance over the no-SAW controls. After 1 min, SAW improves the absorbance by 51%. This trend continues throughout. As the elapsed time is increased from 5 to 15 min, SAW produces a roughly linear improvement in the absorbance, growing to exceed the traditional 1 h no-SAW at 35 °C (red dotted line) after just 15 min of SAW. It is likewise 294% greater (*p* < 0.01) than the absorbance of the no-SAW ELISA run for the same amount of time at a lab temperature of 25 °C. As a result of faster binding, it took only 10 min to achieve the same binding result as the standard 35 °C ELISA after 1 h. After waiting 10 min with SAW, one can obtain an absorbance of twice the ELISA result without SAW for 10 min.

While there is a correlation between the absorbance and the maximum measured temperature of each sample, the absorbance value does not correspond to the maximum temperature value measured while applying SAW, indicating that the effects of the SAW are not solely due to temperature increases alone. From 1 to 5 min of SAW, the maximum sample temperature increases by 5 °C and the absorbance improves from 0.093 to 0.15. However, from 5 to 15 min of SAW, the maximum sample temperature only increases by 1 °C while the absorbance nearly doubles from 0.15 to 0.33.

### Effect of SAW on Antibody–Antigen Binding in Dynabead-Based ELISA

**Figure 4:**
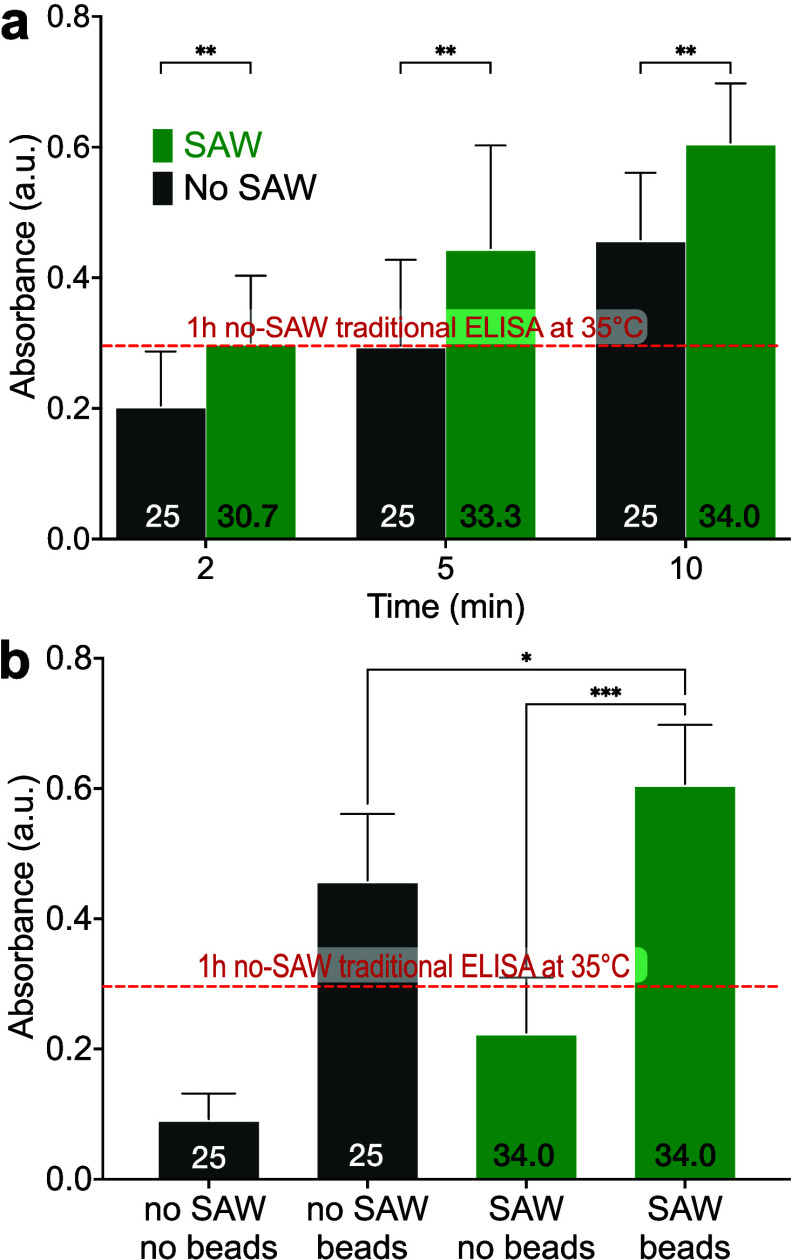
Using a bead-based ELISA a) vastly improves the speed to produce an absorbance comparable to the standard 1-h, no-SAW, 35 °C ELISA protocol (red dashed line) after only 5 min, with 2.5 μL of Dynabeads (30 mg/mL) coated with 200 μL antiprotein A antibody (5 μg/mL) and dilution of the goat antichicken IgY-HRP stock solution to 10 ng/mL in PBS for this experiment. Adding 60 MHz, 192 mm/s, 0.8 W SAW to the bead-based ELISA significantly improves the speed to obtain the same results as the traditional protocol after only 2 min. Comparing the results after 10 min incubation, b) either introducing beads into the system or introducing SAW into the bead-based system significantly improves the absorbance. As before, the maximum temperature of the samples in Celsius is provided at the bottom of each bar. There was no externally applied heating, and the temperature of the samples was at the lab temperature of 25 °C, initially for the SAW-driven samples, and throughout for the no-SAW (control) samples. Error bars represent the standard deviation of 6 independent trials. All data was confirmed to be normally distributed via the Shapiro-Wilk test; *p < 0.05, **p < 0.01, ***p < 0.001.

Functionalized beads are also commonly used in ELISA, providing increased surface area and mixing of the beads in the solution, in turn leading to greater sensitivity and, for that matter, multiplexing capabilities.^61^ Here, we also explored whether SAW can enhance binding in the bead-based ELISA system. Using ferromagnetic 2.8 μm diameter Dynabeads at a concentration of 0.375 mg/mL, we applied SAW at the antibody–antigen binding step for 2 to 10 min. As expected, using Dynabeads produces a faster ELISA: Dynabeads reduces the time required to produce the same absorbance from 1 h (red dotted line) to 5 min (Figure 4a). However, using 60 MHz, 192 mm/s, 0.8 W SAW with the Dynabeads reduces the time necessary to perform the ELISA to just 2 min.

Put another way, in the bead-based approach to ELISA, SAW stimulation is found to significantly (Figure 4b; *p* < 0.05) increase the absorbance compared to the no-SAW, bead-based control after 10 min incubation. Using Dynabeads also significantly improves the absorbance value (Figure 4b; *p* < 0.001). By combining the accelerated binding kinetics from SAW and the transport of the roughened binding surface in the form of Dynabeads through SAW-driven mixing, the absorbance was found to be over six times greater than the no-SAW, no-bead control after 10 min incubation. Moreover, after just 10 min, the combination of SAW and Dynabeads produces an absorbance value twice the 1 h no-SAW, no-bead result (Figure 4a).

### Effect of SAW on Antibody-Well Binding: A Required Coating Step in Traditional ELISA

We have verified that SAW stimulation can significantly enhance the efficiency of antigen–antibody binding, regardless of whether the antigen/antibody was previously bonded to the well or to the beads. The antigen/antibody must first be bound to the well in traditional ELISA, and this takes considerable time in the overall assay. Even if SAW improves the performance of the antigen–antibody binding, the net benefit of doing so is reduced if the antibody-well wall binding still takes a long time. Therefore, we next examine the effect of SAW on the antibody-well binding as a required step in standard ELISA.

**Figure 5:**
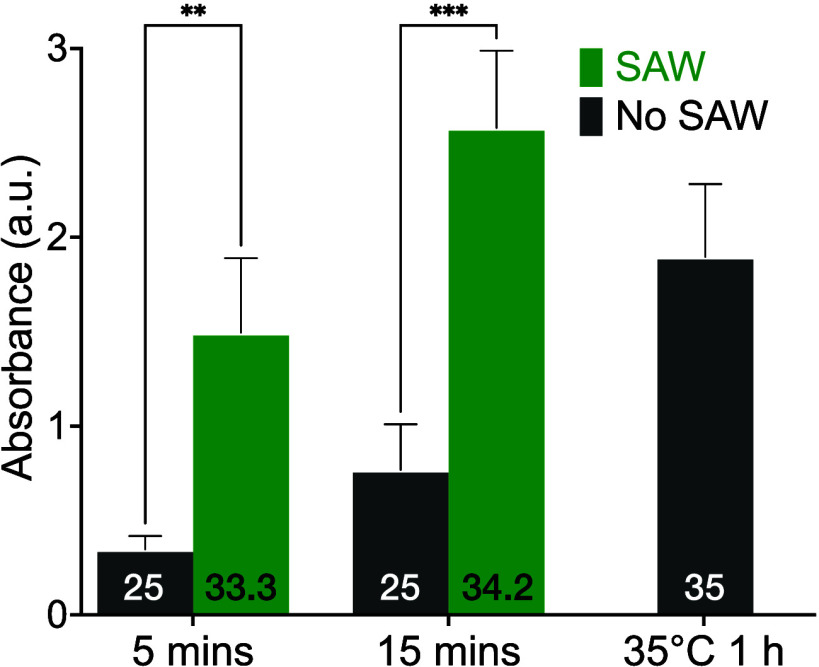
Time course of antibody-well binding with and without 60 MHz, 0.8 W SAW control. 96-well plates were coated with 200 μL of the goat antichicken IgY-HRP (250 ng/mL) in PBS at 35 °C for 1 h, room temperature for 5, 15 min and SAW was applied for the indicated time as 5 and 15 min; TMB was then introduced and incubated without SAW for 5 min. Error bars represent a standard deviation of 5 independent trials; *p < 0.05, **p < 0.01, ***p < 0.001. The maximum temperature of the samples in Celsius is provided at the bottom of each bar. There was no externally applied heating, and the temperature of the samples was at the lab temperature of 25 °C, initially for the SAW-driven samples, and throughout for the no-SAW (control) samples. Comparing the traditional 35 °C, 1-h ELISA protocol shows that the improvement of the ELISA due to SAW is not due solely to heating.

We used goat antichicken IgY-HRP as the surface binding molecule for the well. This allowed us to utilize the HRP-TMB reaction as a measure of binding efficiency. As presented in Figure 5, our findings indicate that SAW stimulation can enhance the antibody-well binding efficiency, producing a two to 3-fold increase in efficiency over the no-SAW control. In comparison to the traditional ELISA protocol of waiting 1 h at 35 °C for binding of the antibody to the well, using SAW produces a comparable absorbance after only 15 min.

The ELISA typically involves two to three steps of antigen–antibody binding and antibody-well binding. Through the application of SAW stimulation, our results indicate a reduction in the overall time required to complete traditional direct sandwich ELISA from approximately 5 h to 1.5 h. Using SAW helps accelerate the binding in both antigen–antibody and antibody-well interactions, thereby streamlining the ELISA process. SAW may also be useful in promoting the speedy deposition of the blocking molecules used to surround the binding sites. However, in our experiments, the use of SAW for this step produced inconclusive results, and so *we retained the traditional blocking protocol* using blocking buffer in PBS at 35 °C for 1 h, as detailed earlier.

### Effect of SAW on ELISA Sensitivity

**Figure 6:**
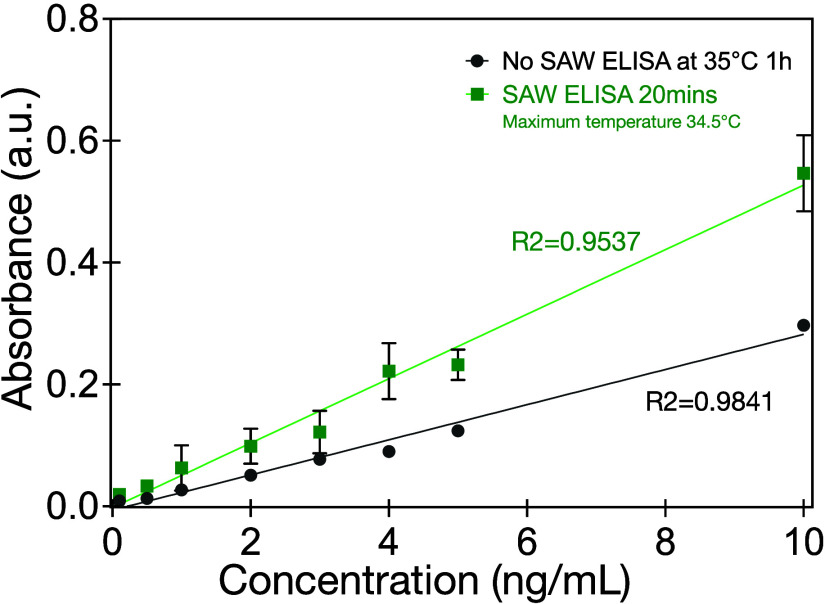
Absorbance indicated binding versus target antigen concentration with and without 60 MHz, 0.8 W SAW. SAW improves the absorbance for the same concentration of the target antigen. Error bars represent the standard deviation of 3 independent trials.

After promising results regarding the time reduction thanks to the SAW, we next focused on improving the sensitivity. We investigated the feasibility of utilizing SAW to facilitate antibody–antigen binding under varying concentrations of the antigen. The goat antichicken IgY-HRP was diluted with PBS to create a concentration range from 0.1 ng/mL to 10 ng/mL, and 0.8 W, 60 MHz SAW was applied for a duration of 20 min to ensure sufficient antigen–antibody binding. For both cases (with and without SAW), results indicate that the binding is linearly dependent upon concentration of the antigen with calculated slopes of 0.0289 and 0.0529 for traditional ELISA for 1 h and SAW for 20 min, respectively (Figure 6). This also demonstrates the improvement in sensitivity of ELISA with SAW stimulation. Notably, under SAW stimulation, the total antigen–antibody binding is found to increase by 83%. Increasing the SAW exposure time to 20 min, causes the total amount of antibody binding to reach equilibrium, the maximum possible in our study. By comparing these results to the traditional 35 °C, 1-h ELISA protocol, it is evident that the improvement in ELISA due to SAW is not simply due to heating, because after 15 min of SAW the sample is at a lower temperature—34.2 °C—yet produces a greater absorbance.

## Discussion

Although there are several acoustically driven ELISA platforms reported in the literature,^62,63^ our approach offers notable advantages. The total time for the standard ELISA protocol is 3 h at 35 °C or 5 h at room temperature. By using SAW as described here, the complete ELISA protocol can be run at room temperature in 1.5 h. It fits into the normal ELISA workflow, using 96-well trays instead of expecting a laboratory to change its operations to accommodate the technology and to deal with the myriad drawbacks of microfluidics in practical laboratory operations.^64^ In our approach, each well can be individually addressed and controlled by a single IDT in a format compatible with multiplexing to drive all 96 wells at once, in a manner that can be performed using commercially available plates without special training. Past acoustofluidic devices require custom fluidic designs and skilled operators accustomed to acoustofluidics and microfluidics, altogether problematic for adoption in a typical biology lab.

By using the standard bead-based assay, the time required to perform the ELISA protocol for a useful sensitivity is much shorter, a well-known result. A total of about 20 min is required for the standard room-temperature assay, including the time to prepare the beads. The beads are ready-to-use with the antibody binding and blocking steps already completed before purchase, and the bead-based kit is expensive as a consequence. Nonetheless, using SAW in the antigen-binding step in combination with the beads halves the required time for this step to produce a similar absorbance value—from 5 to 2 min. To be consistent, we retained the 5 min bead resuspension step required at the beginning of the standard bead-based ELISA protocol. We also used the 1 h blocking step for the 96-well tray per the standard protocol to avoid background noise, but this can be done in advance. In any case, it is likely that the bead resuspension step could simply be eliminated, reducing the total time required for the bead-based ELISA protocol from 20 min to just 2 min.

These improvements are due to effects beyond SAW-driven heating of the samples. We report the maximum temperature achieved during SAW use, and in every case the temperature never reached the 35 °C used for the traditional 1 h ELISA protocol, yet still we see greater absorbance values after a shorter period of time. For example, after 15 min in Figure 3. Moreover, there is no direct correlation between the temperature and absorbance across the SAW ELISA tests. The mechanism must rely in part upon another effect.

Notably, diffusion responsible for aiding the binding in ELISA in the absence of SAW-driven convection is well-known to be slow. Antigens near the well walls will quickly bind, leaving a depletion zone that must be repopulated by additional antigens from the bulk of the fluid sample via diffusion. If the SAW-driven flow was merely laminar, there would be no advantage in driving the flow: the time to bind a given number of antigen molecules from a fluid sample with laminar flow is identical to a fluid sample with no flow at all.^65^ However, fast acoustic streaming^46^ of the sort present in this system produces nonlaminar flow and mixing,^66^ suggesting that a combination of convection-dominated binding phenomena together with the mixing of the sample fluid is a significant reason for the improvement in binding seen with the use of SAW.

## Conclusions

By utilizing acoustic streaming produced by high frequency surface acoustic waves, we developed a time-saving and improved sensitivity ELISA that may be directly applied to standard protocols in use today in biology, chemical tooling, and diagnostic laboratories to reduce the time required for the full protocol from 3–5 to 1.5 h. It does not require any modifications to the existing ELISA protocol. Instead, operators simply place the ELISA plate on top of our device and allow acoustic streaming generated in the fluid sample to facilitate faster and more sensitive detection of the target biomarkers. We demonstrated that SAW can be applied in the antibody-well and antigen–antibody binding steps of the protocol, leaving the blocking step unmodified. One may also combine SAW with bead-based ELISA to reduce the total time required for the antigen–antibody binding step in that protocol from about 10 to 2 min. The total antigen–antibody binding is increased by 83%, thus increasing the detection sensitivity. With its high sensitivity and efficiency, this approach to ELISA may produce important benefits in the development of diagnostic and therapeutic applications wherever ELISA and its derivatives are used.
